# A Key Biomarker lnc-ABCA12-5:2 Compensating Diagnostic Deficiencies for Indeterminate Thyroid Nodules and Genetically Wild-Type Thyroid Malignancies

**DOI:** 10.32604/or.2026.088872

**Published:** 2026-10-28

**Authors:** Xuan Su, Wen-na Xu, Bu Zou, Chan Huang, Cai-yun He, Zu-lu Ye

**Affiliations:** 1 State Key Laboratory of Oncology in South China, Guangdong Provincial Clinical Research Center for Cancer, Sun Yat-sen University Cancer Center, Guangzhou, China; 2 Department of Head and Neck, Sun Yat-sen University Cancer Center, Sun Yat-sen University, Guangzhou, China; 3 Department of Clinical Laboratory, Sun Yat-sen University Cancer Center, Sun Yat-sen University, Guangzhou, China; 4 Department of Pathology, The Sixth Affiliated Hospital, Sun Yat-sen University, Guangzhou, China; 5 Guangdong Provincial Key Laboratory of Colorectal and Pelvic Floor Diseases, The Sixth Affiliated Hospital, Sun Yat-sen University, Guangzhou, China; 6 Department of Molecular Diagnostics, Sun Yat-sen University Cancer Center, Sun Yat-sen University, Guangzhou, China

**Keywords:** lncRNA, fine-needle aspiration, indeterminate thyroid nodule, thyroid cancer, diagnostic efficacy

## Abstract

**Background:** Preoperative fine-needle aspiration (FNA) cytology and conventional genetic testing often fail on indeterminate thyroid nodules and malignancies lacking typical driver mutations. We validated two candidate lncRNAs, lnc-ABCA12-5:2 and lnc-MPEG1-1:1, initially screened in papillary thyroid carcinoma (PTC) tissues, using preoperative FNA and postoperative formalin-fixed paraffin-embedded (FFPE) specimens. **Methods:** Diagnostic performance was assessed in two independent cohorts encompassing diverse Bethesda categories and thyroid carcinoma subtypes. Molecular correlations and clinicopathological associations were also examined. **Results:** Both lncRNAs exhibited excellent diagnostic efficacy for PTC in preoperative FNA samples (area under the curve values: 0.923 and 0.968; sensitivity > 0.85; specificity > 0.90). lnc-ABCA12-5:2 achieved 100% specificity and 100% positive predictive value in Bethesda III–IV nodules, and was an independent predictor alongside BRAF V600E. Integrating lnc-ABCA12-5:2 with BRAF V600E testing raised diagnostic accuracy in indeterminate nodules from 89.5% to 95.0% (95.7% sensitivity, 92.9% specificity). lnc-ABCA12-5:2 positivity increased progressively with higher Bethesda grades (*p*-trend < 0.001), achieved 100% positivity in high-grade aggressive carcinomas, and remained elevated (83.6%) in genetically wild-type malignant nodules. Its expression strongly correlated with aggressive molecular signatures (including TERT, TP53, PI3K/AKT/PTEN alterations), yielding 100% positivity in such cases. *In vitro* cellular functional assays further confirmed that lnc-ABCA12-5:2 overexpression significantly promoted cell proliferation, colony formation, and migration in PTC cell lines. **Conclusions:** lnc-ABCA12-5:2 and lnc-MPEG1-1:1 are robust preoperative biomarkers for PTC. lnc-ABCA12-5:2 fills critical diagnostic gaps in indeterminate and genetically wild-type thyroid malignancies, and correlates with aggressive tumor molecular profiles, underscoring its translational potential for clinical risk stratification.

## 1 Introduction

Thyroid cancer (TC) is the most common endocrine malignancy worldwide, with a steadily rising incidence [1]. Thyroid nodules, another prevalent endocrine-related condition, are also increasing in frequency [2]. While most thyroid nodules are benign, a small proportion are malignant, with papillary thyroid carcinoma (PTC) accounting for the vast majority of cases [3]. Accurate preoperative discrimination between benign and malignant nodules is critical for avoiding unnecessary surgical resection of benign lesions and ensuring timely and appropriate treatment for malignancies [4,5].

Fine-needle aspiration (FNA) cytology remains the gold standard for preoperative evaluation of thyroid nodules and serves as the cornerstone of clinical decision-making [3,5,6]. Nevertheless, inherent limitations of cytological assessment lead to persistent diagnostic bottlenecks [7]. A substantial proportion of nodules are classified as Bethesda III–IV indeterminate lesions, which cannot be definitively diagnosed as benign or malignant [7]. While somatic testing, including BRAF V600E, has markedly improved preoperative diagnostic accuracy, 20–30% of cases are BRAF wild-type [8,9]. Even with expanded somatic variant panels, this subgroup shows no detectable genetic alterations, and malignancy cannot be ruled out. Accordingly, there is an urgent clinical demand for novel, high-efficiency molecular biomarkers to supplement and optimize the preoperative diagnostic workflows for thyroid nodules.

Accumulating evidence has demonstrated that aberrantly expressed long non-coding RNAs (lncRNAs) exhibit high tissue and disease specificity, positioning them as ideal candidate biomarkers for early tumor diagnosis [10,11]. Previously, we screened for differentially expressed lncRNAs between PTC and adjacent noncancerous tissues using lncRNA expression microarrays. From the significantly dysregulated candidates, ten lncRNAs, including five upregulated (lnc-MPEG1-1:1, lnc-ABCA12-5:2, lnc-RXRG-1:10, FAM230B, and lnc-LRRK2-1:1) and five downregulated (lnc-CENPJ-1:4, lnc-AADACL2-1:3, lnc-TCF4-5:18, lnc-ARHGAP10-2:2, and lnc-PDLIM3-5:1), were prioritized for validation. Quantitative real-time PCR robustly confirmed the differential expression of these lncRNAs in both the TCGA-THCA dataset (n = 494 PTC vs. 58 normal samples) and the prospectively collected cohort from Sun Yat-sen University Cancer Center (SYSUCC; n = 73 paired samples). Clinical correlation analyses demonstrated that lnc-MPEG1-1:1 and lnc-ABCA12-5:2 exhibited strong diagnostic performance. A multivariate logistic model incorporating age, T stage, and these two lncRNAs was constructed and presented as a nomogram to predict lymph node (LN) metastasis in the TCGA screening cohort, and it was subsequently validated in the SYSUCC cohort. However, the clinical applicability of these two lncRNAs requires further evaluation, particularly in preoperative FNA samples. Their diagnostic performance for indeterminate nodules and their complementary role in other histological subtypes of thyroid malignancies remain unclear.

In this study, we focused on preoperative clinical diagnosis scenarios and systematically validated the diagnostic efficacy of lnc-ABCA12-5:2 and lnc-MPEG1-1:1 in preoperative FNA specimens. We further assessed whether coexistent chronic autoimmune thyroiditis (CAT) and nodular goiter influenced diagnostic accuracy. Additionally, we explored the complementary diagnostic value of these lncRNAs in BRAF V600E-negative thyroid malignancies, and examined the associations of their expression positivity with cytological malignancy grade, histological subtype, and canonical gene alterations. This study aimed to establish a novel lncRNA-based auxiliary diagnostic model, address the limitations of conventional cytology and genetic testing, and provide new molecular evidence and clinical strategies for precise preoperative diagnosis and risk stratification of thyroid nodules.

## 2 Materials and Methods

### 2.1 Study Population and Sample Collection

The study protocol adhered to the principles of the Declaration of Helsinki and was approved by the Institutional Ethics Committee of Sun Yat-sen University Cancer Center (approval No. B2026-266-01). All patients provided written informed consent at their first visit. This real-world observational study was conducted at SYSUCC, enrolling consecutive patients with thyroid nodules who required preoperative or postoperative molecular evaluation between 01 July 2024 and 01 December 2025. As a retrospective study, ethics approval was obtained before data collection, with additional lncRNA detection performed on residual nucleic acid specimens. Eligible patients were those who underwent either preoperative FNA cytology with BRAF V600E testing, or postoperative pathological assessment of formalin-fixed paraffin-embedded (FFPE) tissue using a 28-gene next-generation sequencing (NGS) panel. Clinicopathological parameters, including LN metastasis, capsular invasion, TNM stage, and coexisting benign thyroid diseases, were collected for all participants. Exclusion criteria were as followings: (1) absence of a definitive pathological or clinical diagnosis; (2) patients with a history of head-and-neck radiotherapy, other systemic malignancies, or incomplete clinical data; (3) poor nucleic acid quality or insufficient nucleic acid quantity for lncRNA detection, defined as a total nucleic acid amount < 1 μg required for cDNA reverse transcription or a GAPDH gene internal control Ct value > 30, indicating possible RNA degradation.

Of the 1159 initially enrolled samples, 328 were excluded according to the above criteria, leaving 831 eligible samples for analysis (Fig. 1). Samples obtained from different puncture or surgical sites of the same patient were included as independent samples in the analysis. For each sample, the diagnostic information was matched to the diagnosis of its corresponding site. Cohort 1 included 541 FNA samples with BRAF V600E detection, which were categorized by cytology diagnosis (Bethesda I–VI) and postoperative diagnosis. Cohort 2 included 290 FFPE samples analyzed by the 28-gene NGS panel, with postoperative diagnoses comprising various histological subtypes of thyroid lesions. Thirteen samples across Cohorts 1 (FNA) and 2 (FFPE) were obtained from the same patients. The two cohorts were analyzed as independent sample sets for distinct purposes and were never merged, so that no sample was double-counted within any analysis. FNA samples were used to assess preoperative diagnostic performance, whereas FFPE samples were used to examine the relationship between lncRNA expression and gene mutation profiles.

**Figure 1:**
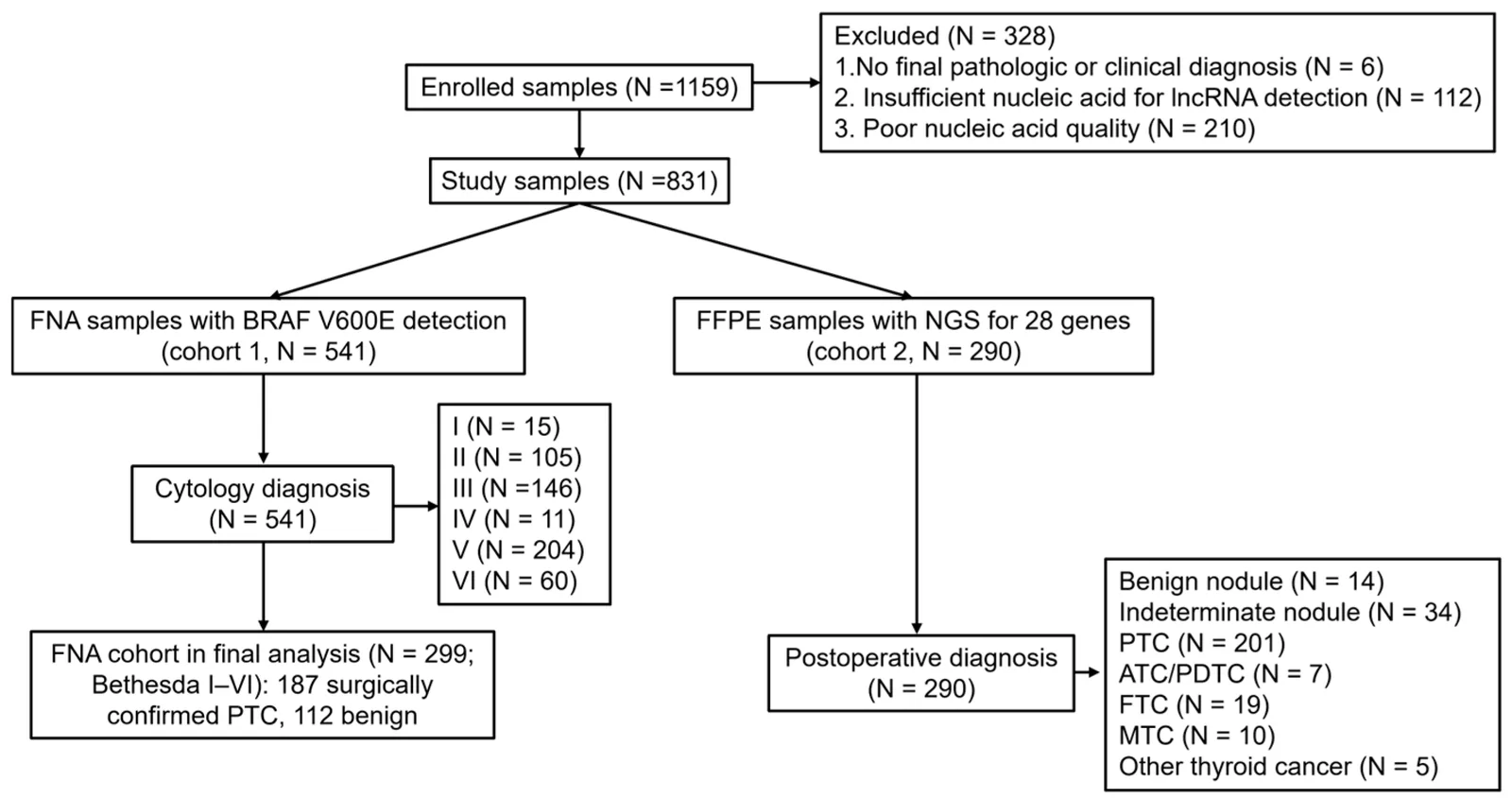
Overview of all enrolled samples in this study. Abbreviations: FNA, fine-needle aspiration; FFPE, formalin-fixed paraffin-embedded; NGS, next-generation sequencing; PTC, papillary thyroid carcinoma; ATC, anaplastic thyroid carcinoma; PDTC, poorly differentiated thyroid carcinoma; FTC, follicular thyroid carcinoma; MTC, medullary thyroid carcinoma.

### 2.2 Nucleic Acid Extraction

Total nucleic acids were extracted from various clinical specimens following the manufacturer’s standardized protocols. FNA samples were preserved in a total volume of 800 μL. Each sample was vortexed for 5–10 s to ensure thorough mixing, and a 400 μL aliquot was then taken for analysis. For preoperative samples, 400 μL of homogenized FNA suspension was processed using an automated total nucleic acid extraction and purification kit (Cat. No. 8.02.0118, Amoy Diagnostics Co., Ltd., Xiamen, China) on an EASY12 automated nucleic acid extraction instrument (Amoy Diagnostics Co., Ltd., Xiamen, China). For postoperative samples, total nucleic acids were isolated from FFPE tissues fixed in 10% neutral-buffered formalin using a commercial nucleic acid extraction kit (Cat. No. RJ006T, Shanghai RIGEN Biotechnology Co., Ltd., Shanghai, China).

Following nucleic acid extraction, the concentration and purity of all nucleic acid samples were assessed. For BRAF V600E and lncRNA detection, samples were measured using a NanoDrop spectrophotometer, with purity evaluated by the OD260/280 ratio; for NGS detection, samples were quantified using a Qubit fluorometer (both from Thermo Fisher Scientific, Waltham, MA, USA). The predefined sample acceptance criteria were as follows: (1) For BRAF mutation detection (Cat. No. 8.01.0151; Amoy Diagnostics Co., Ltd., Xiamen, China), samples with a nucleic acid concentration ≥1.5 ng/μL and an OD260/280 ratio of 1.8–2.0 were considered qualified. (2) For 28-gene NGS testing (Cat. No. RJ013J; Shanghai RIGEN Biotechnology Co., Ltd., Shanghai, China), samples with a nucleic acid concentration ≥2 ng/μL and an OD260/280 ratio of 1.8–2.1 were accepted for library construction and sequencing. (3) For lncRNA detection (Cat. No. RR047A; TaKaRa Biotech, Dalian, China), samples with a total nucleic acid amount >1 μg for cDNA reverse transcription and an OD260/280 ratio of 1.8–2.0 were considered qualified. Only qualified nucleic acid samples were subjected to subsequent molecular detection and data analysis according to the manufacturers’ standard operating procedures.

### 2.3 Real-Time Quantitative PCR (RT-qPCR) for lncRNA

Total RNA was reverse-transcribed into complementary DNA (cDNA) using the PrimeScript™ RT reagent kit (Cat. No. RR047A; TaKaRa Biotech, Dalian, China) in accordance with the manufacturer’s guidelines. A total nucleic acid amount of 1 μg was reverse-transcribed into cDNA in a total reaction volume of 20 μL. The reverse-transcribed reaction was incubated at 37°C for 15 min, followed by 85°C for 5 s for enzyme inactivation. Quantitative PCR was subsequently performed with the SYBR^®^ Premix Ex Taq™ II kit (Cat. No. RR820A; TaKaRa Biotech) on an Applied Biosystems™ 7500 Real-Time PCR System (Foster City, CA, USA). qPCR was performed in a 20 μL total reaction system. Each reaction contained 2 μL cDNA template, forward and reverse primers with a final concentration of 0.4 μM. Thermal cycling conditions were as follows: initial pre-denaturation step at 95°C for 30 s, followed by 40 amplification cycles of 95°C for 5 s and 60°C for 34 s. GAPDH gene served as the internal reference for normalizing the relative expression of target lncRNAs. Relative expression of lncRNA was calculated using the 2^−ΔΔCt^ method. For this clinical cohort, each sample was tested in a single qPCR reaction, and no extra biological or technical replicates were included. The sequences of the primers used in this study were summarized in Supplementary Table S1.

### 2.4 Detection of BRAF V600E and Canonical Gene Alterations

BRAF V600E detection was performed using a Hongshi SLAN-96S PCR instrument (Shanghai Hongshi Medical Technology Co., Ltd., Shanghai, China). BRAF V600E detection was performed based on the TaqMan probe-mediated real-time fluorescent PCR principle. Each PCR run was strictly accompanied by kit-provided positive and negative controls to monitor reaction validity and avoid false results. According to the predefined assay criteria, a sample was defined as BRAF V600E-positive when a typical sigmoidal amplification curve was obtained with Ct < 30; samples without valid amplification signals or with Ct ≥ 30 were classified as negative.

The 28-gene NGS panel was designed to detect multiple genetic alterations associated with thyroid cancer and covered 28 individual genes, supporting the identification of diverse variant types across these target genes. For NGS detection, the input DNA amount for library construction was 20 ng. Sequencing was performed on an Illumina NovaSeq 6000 platform (Illumina Inc., San Diego, CA, USA) with a read length of 150 bp. The mean target sequencing depth was 4000×, and samples that failed to meet the minimum target-region coverage threshold of 50× were excluded from subsequent variant calling. Raw sequencing data were processed using the bioinformatics pipeline provided by the commercial analysis software of the 28-gene panel kit. Briefly, FASTQ files were subjected to quality control, sequence alignment, duplicate removal, and base-quality recalibration. Single-nucleotide variants (SNVs) were analyzed for AKT1, ATM, BRAF, CHEK2, CTNNB1, EIF1AX, EZH1, FGFR1, FLT3, GNAS, HRAS, KIT, KRAS, NRAS, PIK3CA, PTEN, SPOP, TP53, TSHR, and ZNF148. SNVs were retained if they met the predefined thresholds of a minimum variant allele frequency (VAF) ≥ 1% and a supporting read count ≥ 3. Gene fusions were detected for NTRK1, NTRK3, PAX8, PPARG, and ROS1. Gene fusions were confirmed by sufficient split-read evidence, and artifactual fusion events were excluded. Both SNVs and gene fusions were examined for ALK and RET. For TERT mutation detection, only variants located in the TERT promoter region that met the predefined VAF and read-support criteria were considered positive. All candidate variants were further manually reviewed for final interpretation.

### 2.5 Clinicopathological and Cytological Evaluation

All FNA cytological specimens were independently evaluated by two experienced cytopathologists and categorized according to the 2nd edition (2017) of the Bethesda System for Reporting Thyroid Cytopathology (TBSRTC), including Bethesda I (non-diagnostic/unsatisfactory; insufficient cellularity or poor specimen quality), Bethesda II (benign), Bethesda III–IV (indeterminate), Bethesda V (suspicious for malignancy), and Bethesda VI (malignant) categories. When disagreements on diagnosis occurred between two pathologists, the case was submitted for departmental consultation to reach a final diagnosis. Postoperative FFPE specimens were pathologically diagnosed and classified into various subtypes, including PTC, follicular thyroid carcinoma (FTC), medullary thyroid carcinoma (MTC), anaplastic/poorly differentiated thyroid carcinoma (ATC/PDTC), benign thyroid lesions, and low-risk tumors such as non-invasive follicular thyroid neoplasm with papillary-like nuclear features (NIFTP) and thyroid tumor of uncertain malignant potential (UMP).

### 2.6 Cell Culture and Transduction

Four human PTC cell lines (TPC-1 [CRL-2838], KTC-1 [CRL-3242], K1 [CRL-2220], and BCPAP [CRL-7094]) were obtained from the American Type Culture Collection (ATCC, Manassas, VA, USA). All cell lines were authenticated by short-tandem-repeat (STR) profiling and confirmed to be mycoplasma-negative prior to use. Cells were cultured in RPMI-1640 medium (Cat. #11875093, Gibco, Thermo Fisher Scientific, Waltham, MA, USA) supplemented with 10% fetal bovine serum (FBS, Cat. #10099141, Gibco) and 1% penicillin-streptomycin mixture (Cat.#15140122, Gibco) and maintained at 37°C in a humidified incubator with 5% CO_2_.

Short hairpin RNA (shRNA) targeting lnc-ABCA12-5:2 (sh-lnc-ABCA12-5:2) and its negative control were custom-designed and synthesized by HanYi Biosciences Co., Ltd. (Shanghai, China). The shRNA sequences targeting lnc-ABCA12-5:2 are provided in Supplementary Table S1. Stable cell lines were constructed via lentiviral transduction (Tsingke Biotechnology Co., Ltd., Beijing, China). Specifically, BCPAP and KTC-1 cells were transduced with sh-lnc-ABCA12-5:2 lentivirus to knock down lnc-ABCA12-5:2 expression, whereas K1 and TPC-1 cells were transduced with a lentivirus overexpressing lnc-ABCA12-5:2 (pLV3-CMV-lnc-ABCA12-5:2-FLAG-CopGFP-Puro) to upregulate its expression. Four groups of stable cell lines were thus established: K1 and TPC-1 cells overexpressing lnc-ABCA12-5:2 (designated lncABCA12) and their empty vector controls (designated vector), as well as BCPAP and KTC-1 cells with lnc-ABCA12-5:2 knockdown (designated shlncABCA12#1 and shlncABCA12#2) and their corresponding shRNA negative controls (designated shNC). All cellular functional assays were performed in triplicate wells for each cell line.

Briefly, cells were seeded in 6-well plates one day before transduction, with each well containing one cell line, to reach approximately 60% confluency the next day. Culture medium and viral supernatant were mixed at a 1:1 ratio (1 mL each), and 2 μL polybrene was added to the mixture. After 10–12 h of incubation, the medium was replaced. Cells were cultured for at least 48 h; if full confluency was achieved within 48 h and passaged if they reached full confluency within this period. Once cells reached confluency, they were split at a 1:3 ratio and subjected to stable cell selection with 2 μg/mL puromycin. Puromycin selection was terminated once all wild-type control cells had been killed.

### 2.7 Cell Proliferation Assay

Cell proliferative capacity was evaluated using the Cell Counting Kit-8 (CCK-8) assay (Cat. No. HY-K0301, MedChemExpress, NJ, USA). Cells from each group were seeded separately into 96-well plates at a density of 2 × 10^3^ cells per well and cultured under standard culture conditions. At 0, 24, 48, and 72 h after seeding, 10 μL of CCK-8 reagent was added to each well, followed by incubation at 37°C for 2 h. Absorbance was measured at 450 nm using a Varioskan LUX microplate spectrophotometer (Thermo Fisher Scientific, Waltham, MA, USA) to assess cell viability and proliferation. Cell-free blank wells containing culture medium and CCK-8 reagent were included, and their absorbance values were subtracted as background from all experimental readings.

### 2.8 Colony Formation Assay

Transduced PTC cells were digested and resuspended to prepare single-cell suspensions. Then, 500 viable cells per well were seeded into 6-well plates and cultured in complete medium for 7 days. After incubation, the formed colonies were fixed with 1 mL of 4% paraformaldehyde at room temperature for 15 min, washed once with PBS, stained with 1 mL of 0.1% crystal violet (Cat. No. C0121, Beyotime Biotechnology, Shanghai, China) for 10 min, rinsed three times in PBS (5 min per wash), air-dried, and then imaged. After staining, a cell cluster consisting of at least 50 cells and measuring approximately 0.3–1.0 mm in diameter was counted as one colony. Merged cell aggregates were excluded from counting.

### 2.9 Transwell Invasion Assay

Cell invasive ability was evaluated using Transwell chambers (Cat. No. 353097, Corning, NY, USA) with 8-μm pore size, pre-coated with Matrigel matrix (Cat. No. 356255, Corning, NY, USA) for invasion assay. Matrigel was diluted with pre-chilled serum-free medium (Cat. #11875093, Gibco) to a final concentration of 0.5 mg/mL, and 100 μL of the diluted Matrigel was added to the upper chamber and incubated at 37°C for at least 1 h to allow polymerization. Then, 200 μL of serum-free medium (Cat. #11875093, Gibco) was added to the upper chamber for hydration at 37°C for 30 min. After the hydration medium was aspirated from the upper chamber, 600 μL of medium containing 20% serum was added to the lower chamber, and 200 μL of cell suspension (5 × 10^4^ transduced cells) was slowly seeded into the upper chamber, followed by incubation at 37°C for 24 h. Subsequently, the medium was aspirated, and the cells were fixed with 500 μL of 4% paraformaldehyde for 20 min, stained with 500 μL of 0.1% crystal violet for 15 min, washed three times with 1 mL PBS, and the residual liquid in the upper chamber was removed; after air-drying, images were captured using an IX83 inverted optical microscope (Olympus Corporation, Tokyo, Japan).

### 2.10 Wound-Healing Assay

Cell migration ability was assessed using a wound-healing assay. Cells from each group (5 × 10^5^ transduced cells) were seeded into 6-well plates and cultured to form a confluent monolayer, with three replicate wells per group for each cell line. A uniform scratch was made across the monolayer using a sterile 100-μL pipette tip, and debris was removed by gentle washing with phosphate-buffered saline (PBS). After scratching and PBS washing, the cells were cultured in RPMI-1640 medium (11875093, Gibco) supplemented with 1% FBS (v/v, 10099141, Gibco) to attenuate cell proliferation during wound-healing observation. Wound closure was photographed at 0 and 24 h post-scratching under an inverted light microscope (CKX53) to evaluate migration. The wound area of each image was measured using ImageJ software. The wound closure rate was calculated as: wound closure rate (%) = [(Initial wound area − Wound area at time point)/Initial wound area] × 100. All measurements were performed based on image analysis with ImageJ.

### 2.11 Statistical Analysis

The diagnostic performance of each lncRNA for preoperative PTC diagnosis was evaluated by receiver operating characteristic (ROC) curve analysis. The postoperative histopathological diagnosis was used as the standard for malignancy. The area under the curve (AUC) with its 95% confidence interval (CI) was computed. The optimal cutoff value for each lncRNA was determined by maximizing the Youden index (sensitivity + specificity − 1). Based on this cutoff, sensitivity, specificity, positive predictive value (PPV), negative predictive value (NPV), and accuracy were calculated. For subgroups with small sample sizes and AUC values approaching 1.0, AUC and corresponding 95% confidence intervals were calculated via bootstrap resampling with 2000 replicates. The DeLong test was used to compare AUCs among different biomarkers. The Cochran–Armitage trend test was applied to assess the trend of lncRNA positivity across ascending Bethesda cytological categories. Associations between lncRNA expression and clinicopathological features, as well as genetic alterations, were examined using appropriate statistical tests. Multivariate logistic regression analysis was further conducted to evaluate the independent contributions of lncRNAs and other confounding factors. For *in vitro* cellular experiments, all assays were performed in triplicate, and results were presented as mean ± standard deviation (SD). Comparisons between two groups were made using the independent Student’s *t*-test or two-way analysis of variance (ANOVA) as appropriate. All statistical tests were two-sided, and a *p* value < 0.05 was considered statistically significant. For test results yielding *p* values less than 0.001, we presented *p* < 0.001 rather than the exact computed value. All statistical analyses were carried out using R 4.3.2 (The R Foundation for Statistical Computing). The authenticity of this article has been validated by uploading the key raw data onto the Research Data Deposit public platform (https://www.researchdata.org.cn/), with the approval number RDDB2026547982.

## 3 Results

### 3.1 Diagnostic Performance and Optimal Thresholds of Two Hub lncRNAs for PTC in Preoperative FNA Samples

In the FNA cohort, two sequential analyses were conducted. First, to establish optimal cutoffs for predicting malignancy, we compared two extreme groups: cytologically confirmed Bethesda category VI lesions that tested positive for *BRAF* V600E mutation (malignant cases) and Bethesda category II nodules that tested negative for *BRAF* V600E mutation (benign controls). In this setting, the AUC values of lnc-ABCA12-5:2 and lnc-MPEG1-1:1 for diagnosing PTC were 0.923 and 0.968, respectively (Fig. 2). Both biomarkers achieved a sensitivity >0.85 and a specificity >0.90 (Table 1). Their overall diagnostic accuracy exceeded 90%, indicating strong discriminatory ability for the identification of PTC based on preoperative FNA. The optimal ΔCT cutoffs, determined by maximizing the Youden index, were 6.92 for lnc-ABCA12-5:2 and 6.99 for lnc-MPEG1-1:1.

**Table 1:** Optimal cutoff values of the two hub lncRNAs distinguishing malignant and benign nodules.

Methods	Group	Malignant	Benign	AUC (95%CI)	*p* Value	Sensitivity	Specificity	PPV	NPV	Accuracy	Youden Index
In cohort 1: Bethesda VI vs. Bethesda II (N = 157)											
lnc-ABCA12-5:2											
	Positive	51	6	0.923 (0.871, 0.975)	<0.001	0.850	0.938	0.895	0.910	0.904	0.788
	Negative	9	91								
lnc-MPEG1-1:1											
	Positive	58	8	0.968 (0.937, 0.998)	<0.001	0.967	0.918	0.879	0.978	0.936	0.884
	Negative	2	89								
In entire FNA cohort (N = 299)											
lnc-ABCA12-5:2											
	Positive	142	9	0.859 (0.814, 0.904)	<0.001	0.759	0.920	0.940	0.696	0.819	0.679
	Negative	45	103								
lnc-MPEG1-1:1											
	Positive	163	15	0.921 (0.886, 0.956)	<0.001	0.872	0.866	0.916	0.802	0.870	0.747
	Negative	24	97								

**Note: Abbreviations:** AUC, area under the receiver-operating-characteristic curve; CI, confidence interval; PPV, positive predictive value; NPV, negative predictive value.

Second, we extended the evaluation to the entire cohort encompassing Bethesda categories I–VI (Supplementary Table S2), in which all malignant cases were confirmed by definitive postoperative pathological diagnoses. In this full cohort, the AUC values of lnc-ABCA12-5:2 and lnc-MPEG1-1:1 were 0.859 and 0.921, respectively (Table 1). Furthermore, a multivariate logistic regression analysis incorporating the two lncRNAs, *BRAF* V600E mutation status, sex, and age revealed that both lnc-ABCA12-5:2 and *BRAF* V600E were independent predictors of PTC (Table 2). In addition, expression levels of the two lncRNAs were not associated with age, sex, or comorbidities, but lnc-ABCA12-5:2 was significantly upregulated in subgroups with central lymph node metastasis (level VI) and in those with capsular invasion (Supplementary Table S3).

**Table 2:** Multivariate logistic-regression analysis to predict malignancy in the entire FNA cohort (N = 299).

Parameters	OR (95% CI)	*p* Value
Gender (Male vs. Female)	1.88 (0.30, 11.76)	0.498
BRAF (Positive vs. Negative)	880.74 (90.14, 8605.44)^a^	<0.001
Age (>50 vs. ≤50)	1.42 (0.33, 6.15)	0.637
lnc-ABCA12-5:2 (Positive vs. Negative)	28.66 (5.62, 146.18)	<0.001
lnc-MPEG1-1:1 (Positive vs. Negative)	0.80 (0.14, 4.50)	0.798

**Note:** ^a^the wide confidence interval (CI) for BRAF V600E is caused by sparse data and complete separation in the logistic regression model. The OR estimate should be interpreted cautiously. **Abbreviations:** OR, odds ratio; CI, confidence interval.

**Figure 2:**
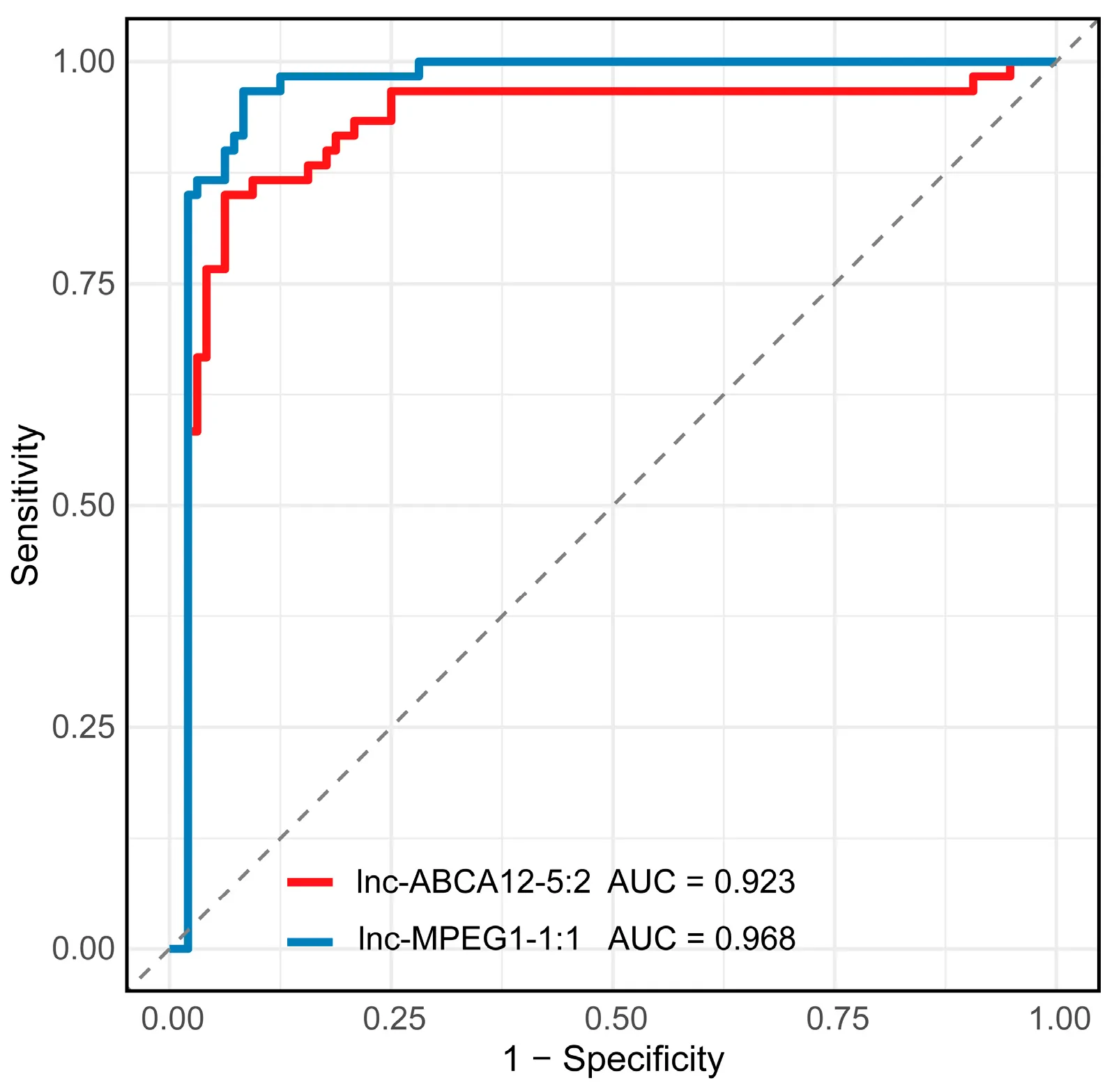
Diagnostic performance of lnc-ABCA12-5:2 and lnc-MPEG1-1:1 for distinguishing malignant nodules (Bethesda VI) and benign nodules (Bethesda II) in Cohort 1. Abbreviations: AUC, area under the curve.

### 3.2 Complementary Diagnostic Value of lnc-ABCA12-5:2 for BRAF V600E-Negative PTC in Cytological Bethesda III–V Thyroid Nodules

Molecular biomarkers were assessed in cytologically indeterminate Bethesda III–V thyroid nodules. Postoperatively confirmed PTC cases were designated as the malignant group, while benign lesions served as controls. Individual detection of lnc-ABCA12-5:2, lnc-MPEG1-1:1, and BRAF V600E mutation yielded comparable AUC values for differentiating malignant from benign nodules (Table 3). Notably, lnc-ABCA12-5:2 demonstrated distinct diagnostic superiority, achieving 100% specificity and a 100% PPV. Among the 28 BRAF V600E-negative nodules, all 9 cases that were positive for lnc-ABCA12-5:2 were ultimately confirmed as PTC, whereas all 13 pathologically verified benign nodules tested negative for this lncRNA (Supplementary Table S4).

**Table 3:** Diagnostic performance of molecular assays for PTC in Bethesda III–V indeterminate nodules within Cohort 1 (N = 153).

Methods	Group	PTC	Benign	AUC (95% CI)	*p* Value	Sensitivity	Specificity	PPV	NPV	Accuracy
lnc-ABCA12-5:2	Positive	95	0	0.842 (0.774, 0.910)	<0.001	0.683	1.000	1.000	0.241	0.712
	Negative	44	14							
lnc-MPEG1-1:1	Positive	101	1	0.828 (0.733, 0.923)	<0.001	0.727	0.929	0.990	0.255	0.745
	Negative	38	13							
BRAF V600E	Positive	124	1	0.910 (0.826, 0.994)	<0.001	0.892	0.929	0.992	0.464	0.895
	Negative	15	13							
lnc-ABCA12-5:2 combined with BRAF V600E	Positive	133	1	0.943 (0.862, 0.989) ^a^	<0.001	0.957	0.929	0.993	0.684	0.954
	Negative	6	13							

**Note:** ^a^we recalculated the 95% confidence interval for the AUC using a bootstrap procedure with 2000 resamples. **Abbreviations:** PTC, papillary thyroid carcinoma; AUC, area under the receiver-operating-characteristic curve; CI, confidence interval; PPV, positive predictive value; NPV, negative predictive value.

A total of 6 PTC cases had negative lnc-ABCA12-5:2 results in preoperative FNA samples; however, retesting of matched postoperative FFPE tumor tissues revealed positive expression in 4 of these cases, suggesting a sampling bias inherent to FNA. One BRAF V600E-negative PTC case remained consistently negative for lnc-ABCA12-5:2 in both preoperative FNA and postoperative FFPE specimens. Further histopathological analysis confirmed the presence of minute tumor foci and an exceptionally low proportion of tumor cells within the whole tissue section. One additional sample yielded invalid results due to nucleic acid degradation. Of note, the following performance metrics were derived from *post-hoc* recalibration using postoperative FFPE retest data, which cannot be obtained in real-world preoperative scenarios; these values should be interpreted only as exploratory descriptive findings and do not represent true preoperative diagnostic performance. Following this *post-hoc* recalibration using the FFPE retest data, exploratory analysis showed that lnc-ABCA12-5:2 achieved an AUC of 0.933 for discriminating benign from malignant lesions in BRAF V600E-negative indeterminate nodules, with a sensitivity of 86.7% and a specificity of 100% (Supplementary Table S4).

### 3.3 Combined Detection of lnc-ABCA12-5:2 and BRAF V600E for Auxiliary Preoperative Diagnosis of PTC

Although detection of lnc-ABCA12-5:2 alone showed no significant superiority over BRAF V600E mutation testing in Bethesda III–IV thyroid nodules, combined detection of the two biomarkers markedly improved diagnostic performance (DeLong’s test, Z = −3.09, *p* = 0.002), yielding an AUC of 0.943, a sensitivity of 0.957, and a specificity of 0.929 (Table 3; the recalibrated combined AUC of 0.957 is presented in Supplementary Table S5). For the combined biomarker analysis, a parallel diagnostic decision rule was applied. A nodule was classified as malignant if either lnc-ABCA12-5:2 was positive or the BRAF V600E mutation was detected, whereas double-negative results were considered benign. This combined panel was further validated in an independent cohort of 146 FNA specimens with definitive postoperative pathological diagnoses (PTC or benign lesions), achieving an AUC of 0.959 (Supplementary Table S6). These findings indicate that lnc-ABCA12-5:2 can act as a valuable adjunct molecular biomarker, complementing BRAF V600E testing to facilitate the auxiliary preoperative diagnosis of PTC.

### 3.4 Diagnostic Value of lnc-ABCA12-5:2 across Different Thyroid Lesions and Its Association with Tumor Aggressiveness

In preoperative FNA specimens, the positivity rate of lnc-ABCA12-5:2 displayed a stepwise increase with ascending Bethesda grades (Fig. 3). The positivity rate remained low in Grades I–III, at 13.3%, 10.5%, and 18.5%, respectively. It rose to 36.4% in Grade IV, sharply increased to 71.1% in Grade V, and peaked at 85.0% in Grade VI. Cochran–Armitage trend analysis confirmed a highly significant positive trend (*p*-trend < 0.001), indicating that lnc-ABCA12-5:2 positivity increases in parallel with increasing cytological malignancy risk.

**Figure 3:**
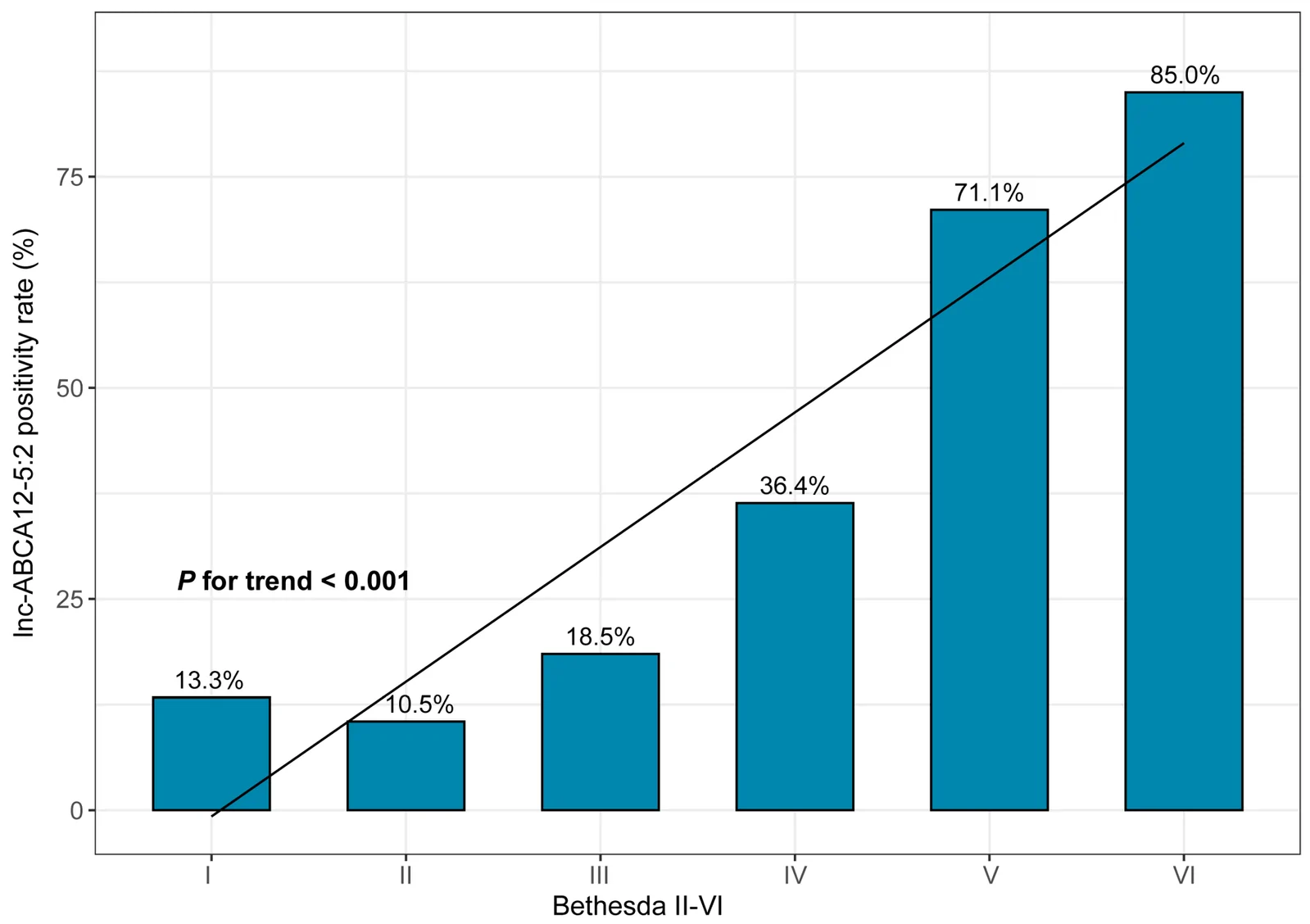
Trend changes in lnc-ABCA12-5:2 positivity rate across cytological Bethesda grades in preoperative FNA samples. Abbreviations: FNA, fine-needle aspiration.

Subsequently, we evaluated lnc-ABCA12-5:2 positivity in postoperative FFPE specimens across seven histopathological categories, including benign nodules, low-risk tumors, and major subtypes of thyroid cancer (Fig. 4). The positivity rate was only 21.4% in benign nodules, suggesting a low false-positive rate, but it increased markedly to 88.2% in low-risk thyroid tumors. Among malignant subtypes of thyroid cancer, the positivity rate reached 89.6% in PTC, 73.7% in FTC, and 70.0% in MTC. Notably, high-grade aggressive tumors, including ATC/PDTC and other rare thyroid malignancies, exhibited 100% positivity. These findings demonstrate that lnc-ABCA12-5:2 is widely expressed across thyroid malignancies, independently of histological lineage, and serves as a stable and sensitive biomarker in FFPE tissues.

**Figure 4:**
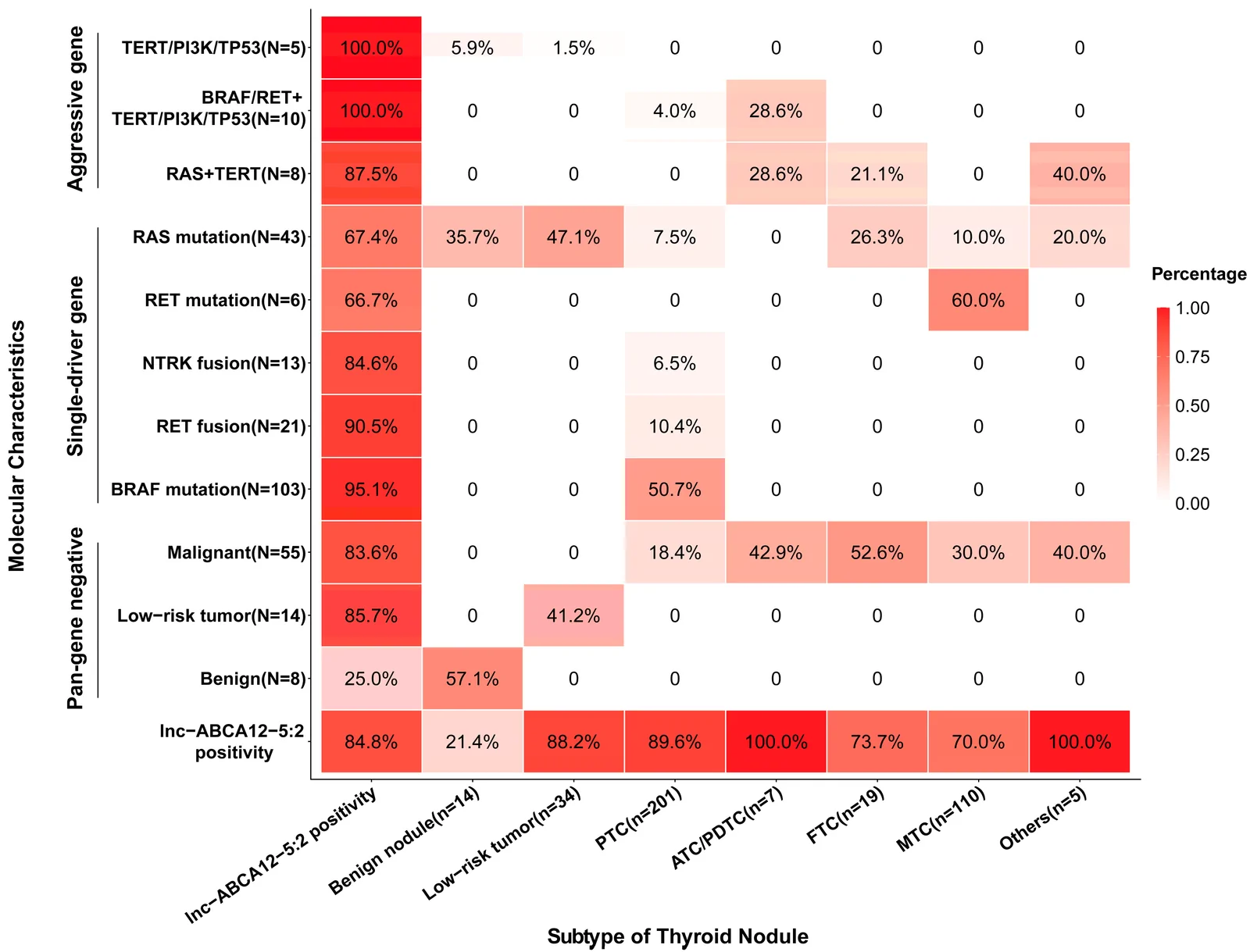
lnc-ABCA12-5:2 positivity rates among distinct thyroid histological subtypes and aggressive molecular phenotypes. Abbreviations: PTC, papillary thyroid carcinoma; ATC, anaplastic thyroid carcinoma; PDTC, poorly differentiated thyroid carcinoma; FTC, follicular thyroid carcinoma; MTC, medullary thyroid carcinoma.

### 3.5 Correlation of lnc-ABCA12-5:2 with Canonical Gene Alterations

The associations between lnc-ABCA12-5:2 positivity and molecular signatures of tumor aggressiveness were summarized across pan-gene-negative, single-driver, and aggressive genetic subgroups (Fig. 4). In PTC nodules harboring single-driver alterations, including BRAF V600E, RET fusions, and NTRK1 fusions, the positivity rates of lnc-ABCA12-5:2 were 95.1%, 90.5%, and 84.6%, respectively. Notably, a 100% positivity rate was observed in nodules carrying concurrent mutations in the above driver genes along with high-aggression genes such as TERT, PI3K/PTEN/AKT and TP53. The same rate was also seen in nodules harboring only these high-aggression mutations. RAS-mutant nodules without other alterations exhibited a positivity rate of only 67.4%. This rate rose to 87.5% when concurrent TERT mutations were present. Strikingly, among 55 malignant thyroid tumors with no detectable mutations in the pan-gene panel, lnc-ABCA12-5:2 still maintained a high positivity rate of 83.6%. This finding substantially fills the diagnostic gap for thyroid nodules lacking identifiable molecular alterations. In contrast, benign nodules with a wild-type molecular background yielded a positivity rate as low as 25.0%. These findings demonstrate that elevated lnc-ABCA12-5:2 expression is strongly correlated with molecular features indicative of tumor aggressiveness in thyroid nodules.

### 3.6 lnc-ABCA12-5:2 Promotes Malignant Phenotypes in PTC Cells

The knockdown and overexpression efficiencies of lnc-ABCA12-5:2 in the established stable cell lines were validated by RT-qPCR (Fig. 5A). We further investigated the effects of lnc-ABCA12-5:2 on malignant phenotypes in PTC cell lines. CCK-8 proliferation assays revealed that silencing lnc-ABCA12-5:2 markedly suppressed cell viability. In contrast, overexpression of this lncRNA accelerated cell growth (Fig. 5B). Consistently, colony-formation capacity was attenuated upon lnc-ABCA12-5:2 silencing and was strengthened after its overexpression (Fig. 5C,D). Wound-healing and Transwell assays further demonstrated that lnc-ABCA12-5:2 knockdown impaired migratory and invasive capacities (Fig. 5E–H). Conversely, lnc-ABCA12-5:2 overexpression significantly promoted PTC cell migration and invasion. These functional experiments confirmed that lnc-ABCA12-5:2 exerts oncogenic effects in PTC cells.

**Figure 5:**
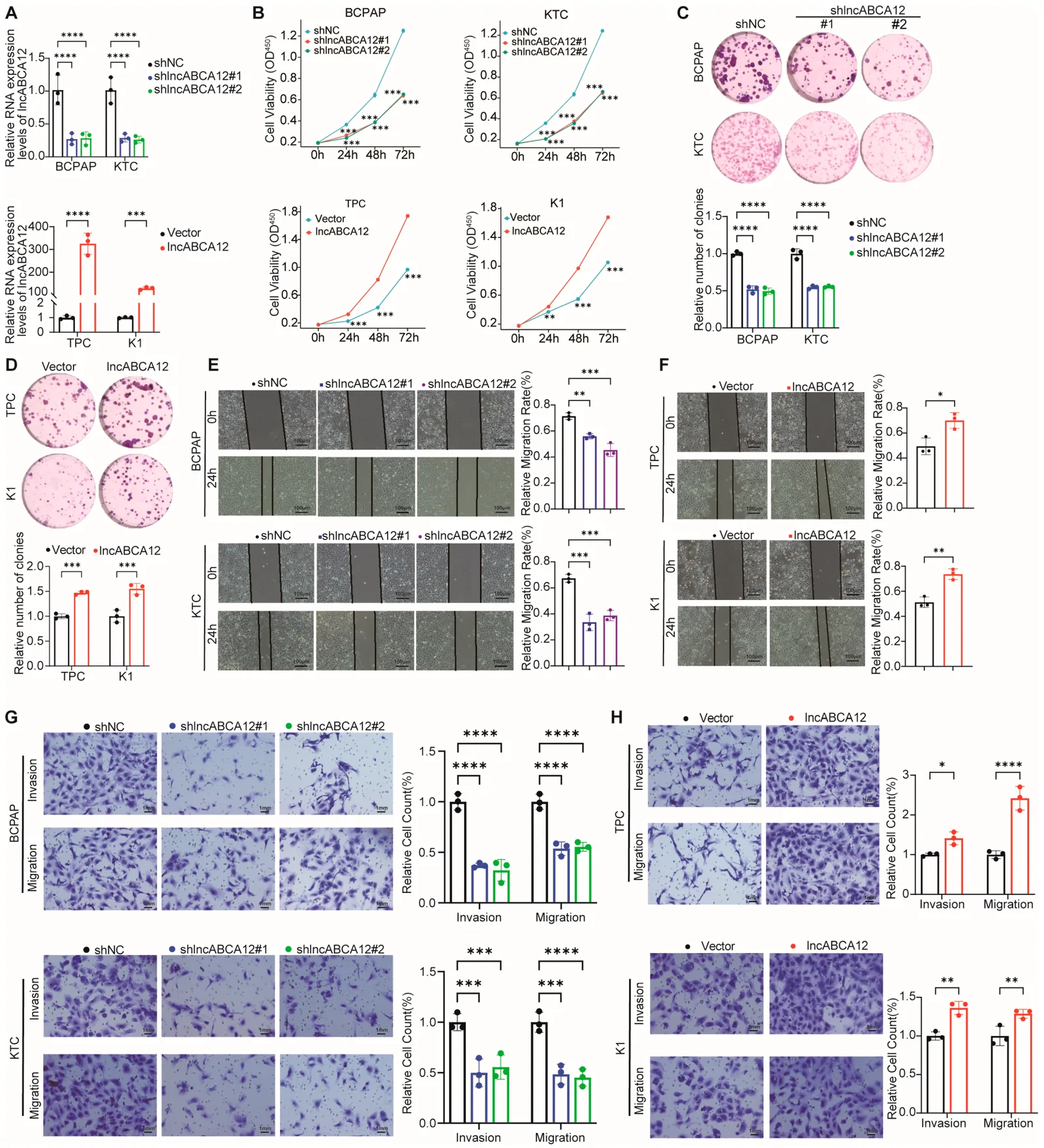
lnc-ABCA12-5:2 promotes malignant phenotypes in PTC cells. (**A**) Quantitative real-time polymerase chain reaction (qRT-PCR) validation of stable lnc-ABCA12-5:2 knockdown and overexpression in PTC cells. (**B**) Cell Counting Kit-8 (CCK-8) proliferation curves for PTC cells with lnc-ABCA12-5:2 knockdown, overexpression, and control. (**C**) Colony formation assay for assessment of clonogenic potential in lnc-ABCA12-5:2-silenced and control PTC cells. (**D**) Colony formation assay for assessment of clonogenic potential in lnc-ABCA12-5:2-overexpressing and control PTC cells. (**E**) Wound healing-assay evaluating cell migration in lnc-ABCA12-5:2-silenced PTC cells (Scale bar: 100 μm). (**F**) Wound-healing assay evaluating cell migration in lnc-ABCA12-5:2-overexpressing and control PTC cells (Scale bar: 100 μm). (**G**) Transwell assay assessing invasion capacities of lnc-ABCA12-5:2-knockdown PTC cells (Scale bar: 1 mm). (**H**) Transwell assay assessing invasion capacities of lnc-ABCA12-5:2-overexpressing and control PTC cells (Scale bar: 1 mm). Data are shown as mean ± SD (standard deviation); grouped comparison by unpaired Student’s *t*-test or two-way analysis of variance (ANOVA); *p < 0.05, **p < 0.01, ***p < 0.001, ****p < 0.0001. Abbreviations: PTC, papillary thyroid carcinoma.

## 4 Discussion

The preoperative diagnosis of thyroid nodules via FNA biopsy remains clinically challenging [5]. In this study, we validated the robust diagnostic performance of lnc-ABCA12-5:2 and lnc-MPEG1-1:1 for preoperative PTC identification using FNA specimens. A key strength of this work lies in the complementary diagnostic value of lnc-ABCA12-5:2 for BRAF V600E-negative PTC within the critical subgroup of Bethesda III–V nodules. Notably, beyond PTC, lnc-ABCA12-5:2 is broadly overexpressed across a spectrum of thyroid malignancies in FFPE tissues, irrespective of histological subtype. Its positivity is also closely linked to aggressive molecular alterations. Furthermore, this lncRNA efficiently discriminates benign from malignant lesions even in the absence of typical molecular aberrations.

Although an increasing number of RNA markers have been shown to differentiate malignant from benign lesions, their clinical translation and practical application in FNA specimens remain extremely limited [12]. To our knowledge, this is the first systematic evaluation of the diagnostic utility of lnc-ABCA12-5:2 and lnc-MPEG1-1:1 using minimally invasive preoperative FNA specimens. The present study verified the robust diagnostic efficacy of these two lncRNAs and established optimal ΔCT cutoff values, providing standardized criteria for clinical interpretation. Consistent with our findings, Liu et al. demonstrated that lnc-MPEG1-1:1 is markedly upregulated in PTC tissues, with higher expression in BRAF V600E-mutated samples [13]. In contrast, lnc-ABCA12-5:2 has not been reported in any published studies. Focusing on lnc-ABCA12-5:2, its expression level in FNA samples increased progressively with Bethesda grade, as confirmed by a significant positive trend in the Cochran–Armitage test. This dose-response relationship links lnc-ABCA12-5:2 positivity to cytological malignancy risk. Moreover, the ΔCT cutoffs derived from FNA specimens remained effective in predicting aggressive phenotypes in FFPE tissues. These findings support lnc-ABCA12-5:2 as a promising auxiliary diagnostic biomarker for PTC. Notably, several cases exhibited inconsistent lncRNA expression between matched FNA and FFPE specimens. Such discrepancies mainly stem from inherent FNA sampling variability, insufficient tumor cellularity in aspirated samples, and mild RNA degradation during clinical specimen processing, which collectively interfere with the accurate detection of lncRNA biomarkers and cause individual diagnostic discordance.

BRAF V600E is the predominant driver mutation routinely used for thyroid nodule diagnosis, yet 20%–35% of PTC cases are BRAF-negative, posing a critical diagnostic challenge [14,15,16]. This necessitates complementary biomarkers to overcome the limitation of single-gene BRAF detection [16]. Our findings demonstrate that combining lnc-ABCA12-5:2 with BRAF V600E detection improves overall diagnostic accuracy for PTC from 89.5% to 95.0% in indeterminate nodules, achieving a sensitivity of 95.7% and a specificity of 92.9%. This enhanced performance may be attributed to the high specificity and PPV of lnc-ABCA12-5:2 in BRAF wild-type malignant lesions. Notably, all lnc-ABCA12-5:2-positive, BRAF-negative nodules were pathologically confirmed as PTC, whereas benign lesions rarely expressed this lncRNA. The minor discordance in lnc-ABCA12-5:2 between FNA and FFPE specimens is likely due to sampling heterogeneity inherent to FNA, which could be mitigated by repeated sampling and combined biomarker testing [17]. Compared with multi-gene panels and NGS-based platforms such as Afirma GSC and ThyroSeq GC, the combined lnc-ABCA12-5:2/BRAF V600E strategy appears to be cost-efficient [17,18,19]. However, these findings are preliminary and require validation in larger independent cohorts and multicenter settings.

lnc-ABCA12-5:2 is not a surrogate marker for BRAF V600E. Instead, it represents an independent biological signal with complementary diagnostic value. Its high expression may indicate broader transcriptional reprogramming. This upregulation can even be observed in molecularly negative PTC subgroups that exhibit aggressive clinical behavior. Multivariate regression analysis identified lnc-ABCA12-5:2 and BRAF V600E as two strong independent predictors of PTC. Moreover, in cytologically indeterminate but BRAF-negative nodules, lnc-ABCA12-5:2 testing provides highly specific evidence of malignancy. Based on these findings, we propose incorporating lnc-ABCA12-5:2 into a tiered molecular testing strategy. For thyroid nodules classified as Bethesda III-V of indeterminate significance, BRAF V600E testing should be performed as the first-line assay. For BRAF-negative cases, additional testing of lnc-ABCA12-5:2 is recommended. Both biomarkers can be assayed using total nucleic acids isolated from FNA specimens without the need for repeated puncture, which ensures favorable technical compatibility. Since the current evidence originates from a single-center retrospective cohort, prospective multicenter investigations are warranted to define its real-world reclassification rate for FNA cytology.

The high positivity rate of lnc-ABCA12-5:2 across thyroid malignancies supports its role as a broad-spectrum biomarker. In FFPE tissues, positivity rates were 89.6% in PTC, 73.7% in FTC, 70.0% in MTC, and 100% in ATC/PDTC and other rare malignancies, versus only 21.4% in benign nodules. Thus, lnc-ABCA12-5:2 is a stable, sensitive biomarker independent of histological lineage. Its expression is higher in aggressive tumors of follicular epithelial origin, including PTC, FTC, PDTC, and ATC, which share lineage-intrinsic molecular alterations [20]. In contrast, MTC arises from parafollicular C cells and is driven by *RET* mutations, with no direct link to follicular-derived cancers [21,22]. At the level of oncogenic pathway activation, all subtypes involve aberrant MAPK and/or PI3K/AKT activation, but with varying dependency and activation modes [23,24]. Future work should identify subtype-specific combinatorial markers and clarify the mechanistic connection between this lncRNA and key oncogenic pathways.

The association between lnc-ABCA12-5:2 expression and aggressive molecular profiles further reinforces its clinical relevance. Positivity rates ranged from 84.6% to 95.1% across single-driver PTC subtypes (BRAF V600E, RET fusion, NTRK1 fusion). Notably, in nodules harboring concurrent driver and high-risk mutations (TERT, PI3K/PTEN/AKT, TP53), or high-risk mutations alone, the positivity rate reached 100%, reflecting a strong correlation with aggressive tumor behavior. Importantly, lnc-ABCA12-5:2 remained positive in 83.6% of mutation-negative malignant tumors, whereas only 25% of wild-type benign nodules tested positive. These findings highlight the potential of lnc-ABCA12-5:2 as a complementary strategy for distinguishing benign from malignant lesions, particularly in molecularly silent cases where conventional genetic testing yields no clinically actionable information.

Cellular functional assays in this study initially confirmed that lnc-ABCA12-5:2 acts as an oncogenic driver in PTC. Knockdown of lnc-ABCA12-5:2 significantly inhibited cell proliferation, clonogenicity, migration, and invasion, while overexpression promoted these phenotypes. Consistent results across four PTC cell lines validated its conserved pro-tumor function and ruled out cell-line-specific bias. These data indicate that lnc-ABCA12-5:2 is required to sustain core malignant behaviors of PTC cells. Further studies should investigate its downstream molecular cascade and prognostic potential.

RAS-like alterations alone indicate neoplastic lesions but do not reliably distinguish benign, malignant, or low-risk entities [25]. Most nodules carrying isolated RAS-like mutations are low-risk neoplasms such as NIFTP and UMP [25], though the spectrum also includes FTC and invasive encapsulated follicular-patterned PTC [6]. In nodules with isolated RAS mutations, lnc-ABCA12-5:2 positivity was 67.4%. When a RAS mutation coexisted with a TERT mutation, the positivity rate rose markedly to 87.5%, further supporting the association between lnc-ABCA12-5:2 expression and elevated malignancy potential in RAS-like tumors. Thus, integrating *RAS*/*TERT* status, lnc-ABCA12-5:2 expression, and histopathology may improve risk estimation and clinical decision-making for *RAS*-altered nodules.

Several limitations of this study should be acknowledged. First, although the diagnostic performance of the two lncRNAs was confirmed in discovery and validation cohorts, all samples were retrospectively collected from a single center, which constitutes internal validation rather than rigorous external validation and may limit the generalizability of our biomarkers. External independent validation in additional patient populations is still required to confirm the generalizability of our findings. Second, the molecular mechanism by which lnc-ABCA12-5:2 promotes malignant phenotypes in thyroid cells remains unclear. While our functional assays demonstrated that this lncRNA promotes PTC proliferation and migration *in vitro*, its upstream regulators and downstream effector pathways require further experimental elucidation. Third, although our data suggest that lnc-ABCA12-5:2 serves as a complementary biomarker for BRAF-negative nodules, the sample size of Bethesda III–V nodules in this study was relatively limited. Future studies with larger cohorts are needed to validate its diagnostic value in this clinically challenging subgroup. Fourth, the precise stratification ability of the combined lnc-ABCA12-5:2 and BRAF V600E testing strategy in FNA specimens requires further confirmation through real-world studies or prospective investigations with larger sample sizes. Addressing these limitations will be essential to facilitate the clinical translation of this promising biomarker panel.

## 5 Conclusions

In conclusion, this study validates the robust preoperative diagnostic efficacy of lnc-ABCA12-5:2 and lnc-MPEG1-1:1 for PTC in FNA specimens. As a novel thyroid biomarker, lnc-ABCA12-5:2 predicts tumor malignancy risk and invasiveness, effectively filling the critical diagnostic gap and significantly improving the diagnostic accuracy for BRAF-negative or molecularly silent malignant thyroid nodules. The proposed combined detection strategy provides a promising preoperative diagnostic approach, and further multicenter and mechanistic studies will facilitate its clinical translation.

## Data Availability

The data used in the preparation of this manuscript are available from the authors on request.

## Supplementary Materials

The supplementary material is available online at https://www.techscience.com/doi/10.32604/or.2026.088872/s1.

## Abbreviations

The following abbreviations are used in this manuscript:

ANOVA	analysis of variance
CI	confidence interval
FNA	fine-needle aspiration
FFPE	formalin-fixed paraffin-embedded
TC	thyroid cancer
PTC	papillary thyroid carcinoma
lncRNAs	long non-coding RNAs
CAT	chronic autoimmune thyroiditis
SYSUCC	Sun Yat-sen University Cancer Center
NGS	next-generation sequencing
cDNA	complementary DNA
GAPDH	glyceraldehyde-3-phosphate dehydrogenase
SNVs	single-nucleotide variants
TBSRTC	the Bethesda system for reporting thyroid cytopathology
FTC	follicular thyroid carcinoma
MTC	medullary thyroid carcinoma
ATC/PDTC	anaplastic/poorly differentiated thyroid carcinoma
NIFTP	non-invasive follicular thyroid neoplasm with papillary-like nuclear features
UMP	uncertain malignant potential
ROC	receiver operating characteristic
AUC	area under the curve
PPV	positive predictive value
NPV	negative predictive value
