# Supplementary material for: A Key Biomarker lnc-ABCA12-5:2 Compensating Diagnostic Deficiencies for Indeterminate Thyroid Nodules and Genetically Wild-Type Thyroid Malignancies
Source: Oncol Res. 2026 Oct 28;34(11):20. doi: 10.32604/or.2026.088872 (PMC13732376; doi:10.32604/or.2026.088872)
Supplement: Supplementary file 1 [file OncolRes-34-88872-s001.zip › TSP_OR_88872-s001.docx]

**Supplementary Table S1 Primer and shRNA sequences used in this study**

| Gene | Sequence (5’- 3’) |
| --- | --- |
| GAPDH (Forward) | TGTTCGTCATGGGTGTGAAC |
| GAPDH (Reverse) | ATGGCATGGACTGTGGTCAT |
| lnc-MPEG1-1:1(Forward) | CTCTTCCACTTAATGCCTGTT |
| lnc-MPEG1-1:1 (Reverse) | GTTTGAATCCCTGCTCCAC |
| lnc-ABCA12-5:2 (Forward) | AGAGCCCGTGTCATTCCT |
| lnc-ABCA12-5:2 (Reverse) | TGCCTCTGCTGGTCTTTC |
| shNC | GGATCCCCTAAGGTTAAGTCGCCCTCGCTCGAGCGAGGGCGACTTAACCTTAGGTTTTTGAATTC |
| shRNA lnc-ABCA12-5:2 #1 | CGTGTCATTCCTCACTATAATT |
| shRNA lnc-ABCA12-5:2 #2 | GCTTTGGATGTACCGATAATT |

**Supplementary Table S2 Distribution of Bethesda categories among malignant and benign lesions in the entire surgical FNA cohort (N = 299)**

| Bethesda categories | Malignant (N = 187) | Benign (N = 112) |
| --- | --- | --- |
| I | 0(0.0%) | 1(0.9%) |
| II | 4(2.1%) | 97(86.6%) |
| III | 24(12.8%) | 12(10.7%) |
| IV | 2(1.1%)^a^ | 0(0.0%) |
| V | 114(61.0%) | 2(1.8%) |
| VI | 43(23.0%)^b^ | 0(0.0%) |

^a^, two preoperative FNA‑classified Bethesda category IV nodules were postoperatively histopathologically diagnosed as one PTC and one FTC; to ensure histological homogeneity of malignant cases, the FTC case was excluded from the PTC diagnostic‑performance analysis for Bethesda III‑V nodules, leaving 153 nodules for final analysis. ^b^, among the overall 60 Bethesda category VI nodules, 43 patients underwent surgical resection at our institution, and all were histopathologically confirmed as PTC postoperatively.

**Supplementary Table S3 Associations between two key lncRNAs and clinicopathological parameters**

| Parameters | N | lnc-ABCA12-5:2 | | lnc-MPEG1-1:1 | |
| --- | --- | --- | --- | --- | --- |
|  |  | Mean±SD | *P* value | Mean±SD | *P* value |
| Sex |  |  |  |  |  |
| Female | 447 | 0.028±0.163 | 0.491 | 0.024±0.079 | 0.730 |
| Male | 94 | 0.016±0.021 |  | 0.021±0.025 |  |
| Age |  |  |  |  |  |
| >=50 | 100 | 0.025±0.076 | 0.946 | 0.037±0.154 | 0.295 |
| <50 | 441 | 0.026±0.161 |  | 0.021±0.032 |  |
| Capsule invasion |  |  |  |  |  |
| Positive | 132 | 0.030±0.026 | < 0.001 | 0.030±0.025 | 0.003 |
| Negative | 55 | 0.017±0.018 |  | 0.021±0.017 |  |
| Concurrent diseases |  |  |  |  |  |
| Negative | 4 | 0.040±0.024 |  | 0.026±0.028 |  |
| Thyroid lymphadenitis | 66 | 0.026±0.026 | 0.274 | 0.022±0.019 | 0.755 |
| Nodular goiter | 114 | 0.026±0.024 | 0.267 | 0.031±0.025 | 0.634 |
| Lymph node metastasis |  |  |  |  |  |
| Positive | 114 | 0.029±0.026 | 0.051 | 0.030±0.026 | 0.050 |
| Negative | 73 | 0.021±0.021 |  | 0.237±0.018 |  |
| Metastatic lymph node compartment |  |  |  |  |  |
| No metastasis | 73 | 0.021±0.021 |  | 0.024±0.018 |  |
| Level VI metastasis | 100 | 0.029±0.027 | 0.048 | 0.031±0.027 | 0.057 |
| Non-level VI metastasis | 14 | 0.026±0.023 | 0.494 | 0.026±0.019 | 0.685 |

Abbreviations: SD, standard deviation.

**Supplementary Table S4 Auxiliary detection of lnc-ABCA12-5:2 in BRAF-negative cytological Bethesda III–V thyroid nodules (n =28)**

| Biomarker | BRAF V600E negative nodules | | AUC(95%CI) | *P value* | Sensitivity | Specificity | PPV | NPV | Accuracy |
| --- | --- | --- | --- | --- | --- | --- | --- | --- | --- |
|  | Malignant | Benign |  |  |  |  |  |  |  |
| **Test results of FNA specimens before adjustment** | | | | | | | | | |
| lnc-ABCA12-5:2 |  |  | 0.800(0.630,0.970) | 0.007 | 0.600 | 1.000 | 1.000 | 0.684 | 0.786 |
| Positive | 9(60.0%) | 0(0.0%) |  |  |  |  |  |  |  |
| Negative | 6(40.0%) | 13(100.0%) |  |  |  |  |  |  |  |
| **After adjustment by retest results of FFPE specimens** | | | | | | | | |  |
| lnc-ABCA12-5:2 |  |  | 0.933(0.833,1.000) | < 0.001 | 0.867 | 1.000 | 1.000 | 0.867 | 0.929 |
| Positive | 13(86.7%) | 0(0.0%) |  |  |  |  |  |  |  |
| Negative | 2(13.3%) | 13(100.0%) |  |  |  |  |  |  |  |

Abbreviations: AUC, area under the receiver‑operating‑characteristic curve; CI, confidence interval; FNA, fine‑needle aspiration; FFPE, formalin‑fixed paraffin‑embedded, PPV, positive predictive value; NPV, negative predictive value.

**Supplementary Table S5 Diagnostic performance of molecular assays in Bethesda III–V indeterminate nodules (Cohort 1, FFPE retest-adjusted, N = 153)**

| Biomarker | BRAF V600E negative nodules | | AUC(95%CI) | *P value* | Sensitivity | Specificity | PPV | NPV | Accuracy |
| --- | --- | --- | --- | --- | --- | --- | --- | --- | --- |
|  | Malignant | Benign |  |  |  |  |  |  |  |
| **After adjustment by retest results of FFPE specimens** | | | | | | | | |  |
| lnc-ABCA12-5:2 combined with BRAF V600E | | | 0.957(0.878,0.999) ^a^ | < 0.001 | 0.986 | 0.929 | 0.993 | 0.867 | 0.98 |
| Positive | 137(98.6%) | 1(7.1%) |  |  |  |  |  |  |  |
| Negative | 2(1.4%) | 13(92.9%) |  |  |  |  |  |  |  |

^a^, we recalculated the 95% confidence interval for the AUC using a bootstrap procedure with 2,000 resamples.

Abbreviations: AUC, area under the receiver‑operating‑characteristic curve; CI, confidence interval; FFPE, formalin‑fixed paraffin‑embedded, PPV, positive predictive value; NPV, negative predictive value.

**Supplementary Table S6 Validation of diagnostic efficacy of lnc-ABCA12-5:2 combined with BRAF V600E in an independent FNA cohort (n = 146)**

| Method |  | Malignant | Benign | AUC (95%CI) | *P* value | Sensitivity | Specificity | PPV | NPV |
| --- | --- | --- | --- | --- | --- | --- | --- | --- | --- |
| lnc−ABCA12−5:2 combined with BRAF V600E | | | | | | | | | |
|  | Positive | 47 | 6 | 0.959 (0.923,0.995) | < 0.001 | 0.979 | 0.939 | 0.887 | 0.989 |
|  | Negative | 1 | 92 |  |  |  |  |  |  |

Abbreviations: AUC, area under the receiver‑operating‑characteristic curve; CI, confidence interval; PPV, positive predictive value; NPV, negative predictive value.
